# Health inequalities among people with disabilities: an umbrella review and evidence synthesis

**DOI:** 10.1016/j.eclinm.2025.103675

**Published:** 2025-12-18

**Authors:** Tracey Smythe, Sara Rotenberg, Maureen Moyo-Chilufya, Jane Wilbur, Hannah Kuper

**Affiliations:** a International Centre for Evidence in Disability, London School of Hygiene and Tropical Medicine, London, UK; b Department of Health and Rehabilitation Sciences, Faculty of Medicine and Health Sciences, Stellenbosch University, Cape Town, South Africa

**Keywords:** People with disabilities, Disabled people, Health, Inequality, Health outcome, Umbrella review

## Abstract

**Background:**

People with disabilities frequently experience poorer health than others in the population, yet the extent of this health gap is unknown. We undertook an umbrella review of meta-analyses to assess the amount, strength and quality of the evidence of the association between disability and a broad range of health outcomes.

**Methods:**

We searched Cochrane Library, EMBASE, Medline, PsycINFO and Health Evidence to identify meta-analyses of quantitative studies, published January 1, 2000 to February 3, 2025, in any language. We included systematic reviews with meta-analyses that compared health outcomes between people with and without disabilities, across all study settings and geographical locations. Two reviewers assessed study eligibility and extracted data. We assessed risk of bias using the AMSTAR2 tool and evaluated the strength of evidence for each meta-analysis according to the Fusar-Poli and Radua criteria. We narratively described the association between disability and health outcomes, categorised according to ICD-11 categories. This study is registered with PROSPERO, CRD42025645729.

**Findings:**

The search generated 11,221 unique records, of which 58 systematic reviews that included meta-analyses were included. Together, these reviews drew on 1409 primary studies from 77 countries and produced 132 separate meta-analyses that evaluated 16 health outcomes. Overall, most systematic reviews were of moderate to low quality. Intellectual and developmental disabilities accounted for the largest share of the meta-analyses (n = 60, 45%). One-third of associations (n = 45, 34%) showed convincing or highly suggestive evidence linking disability to adverse health outcomes. The majority of meta-analyses (n = 113, 86%) found statistically significant and positive associations. No studies that examined disability in relation to diseases of the blood, diseases of the immune system, diseases of the musculoskeletal system or conditions related to sexual health were identified.

**Interpretation:**

People with disabilities are a diverse group, yet share the common experience of markedly worse health than their peers without disabilities. The evidence base is constrained by limited measurement of subjective health outcomes and definitions of disability that may not capture contextual factors. Consequently, the true association of disability and poor health outcomes may be underestimated. Health inequities experienced by people with disabilities necessitate health system reforms with efforts to embed inclusion and address social determinants of health.

**Funding:**

The 10.13039/501100000272National Institute for Health and Care Research, the Programme for Evidence to Inform Disability Action grant from the Foreign, Commonwealth and Development Office, the 10.13039/100000910Conrad N. Hilton Foundation.

**Research in context**

**Evidence before this study**

There is growing evidence that people with disabilities experience health inequities due to their poorer social determinants of healthand worse access to healthcare. We searched PubMed on February 2, 2025 for reviews published in the last 15 years about the prevalence of poor health among people with disabilities compared to those without. We used the following combination of keywords: “disability” AND (“health”) AND “meta-analysis”. Only two scoping reviews attempted to examine the broader relationship between disability and health: one was a “rapid scoping review” with an unclear search strategy, and the other focused on health system barriers and interventions. This search highlights a key gap which our review seeks to fill, namely the lack of a comprehensive, methodologically robust synthesis of meta-analyses examining the full scope of health inequalities experienced by people with disabilities.

**Added value of this study**

Our umbrella review of meta-analyses exploring the association of disability and health outcomes allowed assessment of the amount, strength and quality of the evidence. We showed that people with disabilities consistently experience health disadvantages across ages and diseases. People with disabilities generally experience twice the frequency of health conditons based on the outcomes where the research evidence is strongest: infectious disease, perinatal, metabolic and mental-health outcomes. Yet evidence is sparse for some demographics (e.g. multiple or age-related disabilities) and health outcomes (e.g. diseases of the blood, diseases of the immune system, diseases of the musculoskeletal system or conditions related to sexual health). This information helps to improve our understanding of the potential pathways that targeted interventions and health system improvements can address.

**Implications of all the available evidence**

People with disabilities experience stark health inequities, which shows that health systems are not currently meeting their needs. There is urgent need for disability-inclusive health policies and practices that address systemic barriers and promote equitable access to care and to close the gap in life expectancy and other health outcomes. Furthermore, by focussing on objective health outcomes and definitions of disability that may not capture contextual factors, the findings may underrepresent the full extent and complexity of health inequities experienced by people with disabilities. Health systems need to strengthen to address health gaps for people with disabilities. There are specific evidence gaps that need to be filled for the association of disability and health status, particularly in relation to blood, immune, musculoskeletal, and sexual health conditions.

## Introduction

There are 1.3 billion people with disabilities globally and, on average, they experience worse health than their peers without disabilities.^1^, ^2^, ^3^, ^4^ People with disabilities include those who have *“long-term physical, mental, intellectual or sensory impairments which in interaction with various barriers may hinder their full and effective participation in society on an equal basis with others.”*^5^ People with disabilities are extremely diverse, yet they share commonalities that increase their vulnerability to poor health. First, they often experience adverse social determinants of health, such as poverty^6^ and lower education.^7^ Second, people with disabilities by definition have an underlying health condition or impairment that can also lead to further conditions. For example, people with Down syndrome are predisoposed to certain cancers^8^ and heart defects.^9^ Third, health system failures hamper the ability of people with disabilities to access timely and high quality services, such as inaccessible health services, limited training of health workers around disability, and lack of availability of rehabilitation or specialist services.^4^ These pathways contribute to stark health gaps. For instance, two recent systematic reviews of global and low-and middle-income country (LMIC) data showed that mortality was over twice as high among people with disabilities than people without disabilities, leading to a 14-year shorter life expectancy.^1^^,^^2^

There is emerging evidence that a large portion of these gaps is avoidable through health system improvements.^10^^,^^11^ For example, the Confidential Inquiry in England and Wales showed that people with intellectual disabilities die 13 years earlier among men and 20 years earlier among women, and that 37% of deaths were avoidable by good quality healthcare among people with intellectual disabilities compared with 13% in the general population.^10^ While some health differences may be attributable to underlying impairments or conditions, many are shaped by modifiable factors related to health systems and social determinants. It is important to distinguish between health inequalities, the measurable differences in health outcomes between groups, and health inequities,^12^ which refer to *“differences in health which are not only unnecessary and avoidable but, in addition, are considered unfair and unjust.”*^13^ Consequently, this paper reports on health inequalities between people with and without disabilities, and in the discussion we consider to what extent these inequalities may reflect underlying health inequities. Up-to-date estimates of the extent of these health inequalities are essential to raise awareness and inform policy initiatives, service planning, resource allocation, and research priorities.^4^^,^^14^ Moreover, the World Health Organisation (WHO) recommends routine equity analyses of health programmes in order to identify which specific groups are being left behind.^15^ A number of studies have reviewed the health inequalities experienced by people with disabilities, largely focused on association of a specific impairment and/or a single health outcome. Nevertheless, they demonstrate the poorer health outcomes experienced by people with disabilities across the lifecourse. For example, reviews show higher frequency of adverse maternity outcomes among women with disabilities,^16^ higher prevalence of malnutrition among children with disabilities,^17^ and more frequent dementia among people with hearing loss.^18^ However, there is a lack of synthesised evidence on whether people with disabilities overall experience a higher frequency of poor health outcomes, and consequently on the extent of the health inequalities and potential inequities facing this group.

The aim of this study is to undertake an umbrella review^19^^,^^20^ of meta-analyses to assess the amount, strength and quality of the evidence of the association between disability and a broad range of health outcomes, and to consider whether this association varies by disability type or health outcome.

## Methods

### Search strategy and selection criteria

We undertook an umbrella review to examine the association between disability and a broad range of health outcomes. The protocol for this review was registered in the International Prospective Register of Systematic Reviews (PROSPERO), reference number CRD42025645729 and was reported according to the Preferred Reporting Items for Overviews of Reviews (PRIOR) reporting guidelines.^21^ This statement was considered more suited for an umbrella review than the Preferred Reporting Items for Systematic Reviews and Meta-Analyses (PRISMA), as it provides for a descriptive summary of quantitative results.

We systematically searched Cochrane Library, EMBASE, Medline, PsycINFO, and Health Evidence from January 1, 2000, to February 3, 2025, to identify available systematic reviews with meta-analysis on the association of disability and health outcomes. There was no restriction on the language or geographical location of publication. We included Medical subject heading (MeSH) terms and keywords with the combination of ‘disability’ AND ‘health outcome’ AND ‘meta-analysis’ (Supplementary Table S1—example of search strategies). We supplemented our database searches with reference tracing.

Disability and people with disabilities were defined by the United Nations Convention on the Rights of Persons with Disabilities^5^ and further conceptualised by the International Classification of Functioning, Disability, and Health Model,^22^ which provides a complementary biopsychosocial framework to classify functioning, activity limitations, and participation restrictions. This definition therefore includes people with specific conditions commonly associated with a higher likelihood of disability (e.g., spina bifida and schizophrenia), those with specific impairments (e.g., visual, hearing, and physical impairments), and those identified based on functioning, activity limitations, or participation restrictions, often measured through self-report instruments (eg, Washington Group questions and activities of daily living scales). No quantitative duration of disability was required. Disability could be self-reported, physician diagnosed, or assessed with a standardised tool.

Health outcomes were defined as a health condition that was measured through prevalence, incidence, or mortality, and were either self-reported, physician diagnosed, or defined through a standardised tool.

Systematic reviews were eligible for inclusion if they examined the relationship between disability and a health outcome, and conducted a meta-analysis that included at least three primary studies of any epidemiological quantitative design (e.g. cohort, case–control, survey). Meta-analyses were required to include a comparator group–either the general population or population without disabilities. Any study setting was eligible, including community- and hospital-based studies.

Systematic reviews were excluded if the group all had a specific non-disability condition (e.g. people who are malnourished, children who are born preterm or an older frail population). Exclusion criteria for some measures of disability included: mild functional impairment (e.g., visual acuity ≥20/40, cognition as measured by the mini mental state examination ≥21, Washington group questions that reported “some” difficulty, only one functional limitation in activities of daily life), depression alone, frailty alone, specific disease that may include mild stages of the condition (e.g. Alzheimer's, Parkinson's) and disability as a continuous measure (i.e., expressing disability severity on a scale without a defined cut-off to classify individuals as having or not having a disability).

Health outcomes were not eligible if they related to medical procedures or clinical decision-making (e.g. caesarean section), threatened issues that had not occurred (e.g. threatened preterm labour, suicidal ideation), or mild/transient conditions (e.g. dental caries only, headache, allergic conditions such as rhinititis and food allergies). Where studies reported overall cancer estimates and individual cancer type data, only the overall estimate was included. We excluded studies on healthcare access (e.g. cancer screening, participation in prevention programmes) and where the outcome was self-reported quality of life.

Two authors (TS, SR) performed different aspects of the searches, and independently undertook title/abstract screening and full-text assessments. Discrepancies were resolved through discussions among authors or with a third author (HK).

### Data analysis

Data were extracted on an excel spreadsheet that was piloted on three studies. Data were extracted by one author (TS) and checked by a second author (SR). Extracted information included: 1) publication characteristics (author, title, year of publication, health outcome, countries of included meta-analyses), 2) characteristics of participants included in the meta-analyses (age, sex, disability), 3) an overall effect-size and 95% CI (eg, HR, OR, RR), and 4) study design (number of studies within the meta-analysis and participants that each effect size represented, corresponding p-values and heterogeneity (I^2^)).

One author (SR) evaluated the quality of each systematic review with the A Measurement Tool to Assess Systematic Reviews (AMSTAR2) tool (available at https://amstar.ca/Amstar-2.php).^23^ The AMSTAR2 questionnaire has 16 criteria and requires reviewers to respond with a “Yes” or “Partial Yes” or “No” or “No Meta-analysis” option. The systematic reviews were consequently categorised as: High (zero or one non-critical weakness); Moderate (more than one non-critical weakness); Low (one critical flaw with or without non-critical weaknesses); and Critically Low (more than one critical flaw with or without non-critical weaknesses) quality.^23^

We categorised the strength of evidence for each individual meta-analysis of the associations disability and health outcomes with the Fusar-Poli and Radua methodology,^24^ previously applied in umbrella reviews.^25^^,^^26^ The strength of evidence was determined for each effect size based on the sample size, p-value, heterogeneity and presence of publication bias, and classed as “convincing”, “highly suggestive”, “suggestive”, “weak” or “non-significant” (Supplementary Table S2). Meta-analyses that did not report on the number of people with disabilities were scored as meeting the criteria if the overall sample size was ≥100,000. When p-values were not reported, we derived the p-value from the effect size and 95% CI in RStudio.

### Data analysis

All analyses were undertaken in RStudio (version 2024.09). We summarised the key features of the included systematic reviews and meta-analyses using descriptive statistics. We identified duplicate primary studies across reviews by comparing populations and outcomes. When substantial overlap was present, the most recent and comprehensive review was retained. As a result, discrepant data between overlapping reviews did not arise. Methodological differences between reviews were described qualitatively. We reported results by health outcome (ICD-11 code or all-cause mortality) and outcomes within each disability category. Meta-analyses reported effect sizes as risk ratios, odds ratios and mean differences. To facilitate comparison of effect sizes, we converted effect sizes derived from continuous outcomes into odds ratios, following established methods.^27^ Specifically, the log OR was calculated as standardized mean difference (SMD) multiplied by π/√3 (approximately 1.81), and exponentiated to obtain the OR. For studies reporting mean differences, SMDs were derived by dividing the MD by the pooled standard deviation, and then converted to ORs using the same formula. We explored the distribution of meta-analyses using a choropleth map^28^ and we mapped the absolute number of meta-analyses per country. We cross tabulated the disability category with ICD-11 code to show the gaps in meta-analyses. Evidence levels were represented by colour coded, size-scaled bubbles.

The original literature search covered publications up to 1 February 2025. To ensure currency, we updated the search on 22 October 2025 using the same databases (Cochrane Library, EMBASE, Medline, PsycINFO, and Health Evidence) and search strategies (Supplementary Table S1).

### Role of the funding source

The funder of the study had no role in study design, data collection, data analysis, data interpretation, or writing of the report. All authors had full access to the data in the study, and TS and HK had final responsibility for the decision to submit for publication.

## Results

**Fig. 1:**
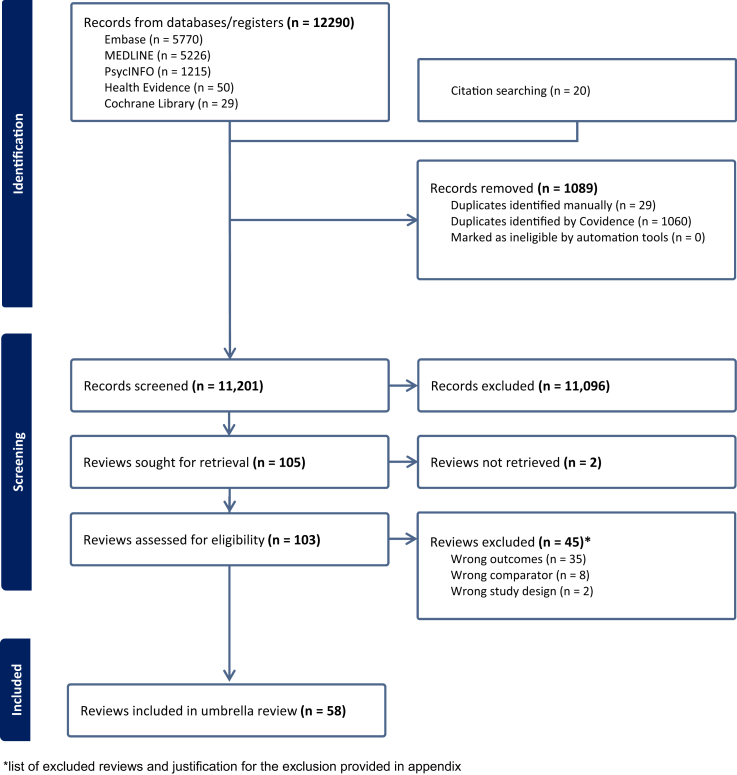
Flow diagram of the systematic review selection process. PRISMA-style flow diagram that illustrates the identification, screening, eligibility assessment, and inclusion of systematic reviews and meta-analyses in the umbrella review. The boxes show the number of records at each stage of selection, with reasons for exclusion provided for full-text reviews.

The database and manual search identified 12,290 records, of which 11,096 were excluded after title and abstract review (Fig. 1). A further 45 records were excluded after reading the full text (reasons for exclusion of full texts described in Supplementary Table S3). Excellent inter-rater reliability for both abstract and full text review was confirmed (κ ≥ 0.75). Subsequently, we identified 58 systematic reviews, which included 132 eligible meta-analyses with data from 1409 primary studies.^1^^,^^2^^,^^16^, ^17^, ^18^^,^^29^, ^30^, ^31^, ^32^, ^33^, ^34^, ^35^, ^36^, ^37^, ^38^, ^39^, ^40^, ^41^, ^42^, ^43^, ^44^, ^45^, ^46^, ^47^, ^48^, ^49^, ^50^, ^51^, ^52^, ^53^, ^54^, ^55^, ^56^, ^57^, ^58^, ^59^, ^60^, ^61^, ^62^, ^63^, ^64^, ^65^, ^66^, ^67^, ^68^, ^69^, ^70^, ^71^, ^72^, ^73^, ^74^, ^75^, ^76^, ^77^, ^78^, ^79^, ^80^, ^81^

The vast majority of systematic reviews were published between 2021 and 2025 (n = 47, 81%). More than half of the systematic reviews reported one health outcome (n = 36, 62%). The quality of the 58 systematic reviews included was generally moderate to low based on the AMSTAR2 scoring (Supplementary Table S4), largely due to failure to examine or address publication bias in the meta-analysis. In total there were 132 meta-analyses included in the 58 systematic reviews. Meta-analyses were undertaken for 15 health outcomes classified under ICD-11 categories, plus an additional outcome of all-cause mortality. The most commonly assessed health category was endocrine, nutritional or metabolic diseases (e.g. diabetes mellitis, obesity) (n = 25, 19%) followed by diseases of the circulatory system (e.g. hypertensive disorders, myocardial infarction) (n = 20, 15%). Notably, there were no eligible meta-analyses that examined diseases of the blood, diseases of the immune system, diseases of the musculoskeletal system or conditions related to sexual health in relation to disability (Table 1). Intellectual and developmental disabilities was the focus of 60 (45%) of the meta-analyses, followed by psychosocial impairments (n = 38, 29%). Eight meta-analyses (6%) focused on all impairment types. More than half of the meta-analyses examined the association of health outcomes for all ages (n = 77, 58%), with some focused on children (n = 22, 17%), and few on older adults (n = 3, 2%). Only 24 (18%) meta-analyses examined health outcomes separately for women with disabilities. Nineteen (14%) meta-analyses included between three and five studies, whilst 34 meta-analyses (26%) included more than 50 studies. The majority of meta-analyses included more than 1 million participants (n = 89, 67%).

**Table 1:** Characteristics of included systematic reviews and meta-analyses.

Category and description		Frequency
Included systematic reviews		N = 58
Date of publication	2000–2005	0%
	2006–2010	0%
	2011–2015	4%
	2016–2020	15%
	2021–2025	81%
Number of outcomes reported per study	1	62%
	2	16%
	3	7%
	4	3%
	5+	12%
Individual meta-analyses in included systematic reviews		N = 132
Health outcome (ICD-11 category) reported per meta-analysis	Certain infectious or parasitic diseases	7%
	Neoplasms	2%
	Diseases of the blood or blood-forming organs	0%
	Diseases of the immune system	0%
	Endocrine, nutritional or metabolic diseases	19%
	Mental, behavioural or neurodevelopmental disorders	13%
	Sleep-wake disorders	0%
	Diseases of the nervous system	2%
	Diseases of the visual system	2%
	Diseases of the ear or mastoid process	0%
	Diseases of the circulatory system	15%
	Diseases of the respiratory system	7%
	Diseases of the digestive system	3%
	Diseases of the skin	2%
	Diseases of the musculoskeletal system or connective tissue	0%
	Diseases of the genitourinary system	0%
	Conditions related to sexual health	0%
	Pregnancy, childbirth or the puerperium	2%
	Certain conditions originating in the perinatal period	10%
	Developmental anomalies	0%
	Symptoms, signs or clinical findings, not elsewhere classified	1%
	Injury, poisoning or certain other consequences of external causes	5%
	External causes of morbidity or mortality	5%
	Factors influencing health status or contact with health services	0%
	Codes for special purposes	0%
	Traditional Medicine Conditions–Module I	0%
	Mortality^a^	5%
Disability domain	All	6%
	Hearing	7%
	Intellectual and developmental	45%
	Neurological	2%
	Physical	2%
	Psychosocial	29%
	Cognitive	0%
	Visual	7%
	Multiple domains	2%
Age group	All ages	58%
	Adults only (any >18 years)^b^	23%
	Older adults only (>60 years)	2%
	Children only (0–18 years)	17%
Sex	Males and females	82%
	Males only	0%
	Females only	18%
Number of studies in meta-analysis	3–5	14%
	6–10	16%
	11–20	17%
	21–30	14%
	31–40	12%
	41–50	1%
	50+	26%
Sample size per meta-analysis	<50,000	12%
	50,001–100,000	1%
	100,001–500,000	6%
	500,001–1,000,000	4%
	>1 million	67%
	Not reported	9%
Classification of evidence of meta-analyses	Convincing	7%
	Highly suggestive	27%
	Suggestive	20%
	Weak	31%
	Non-significant	15%

a 7 meta-analyses reported all-cause mortality, not an ICD-11 category.

b Of these meta-analyses, n = 24 reported women only.

**Fig. 2:**
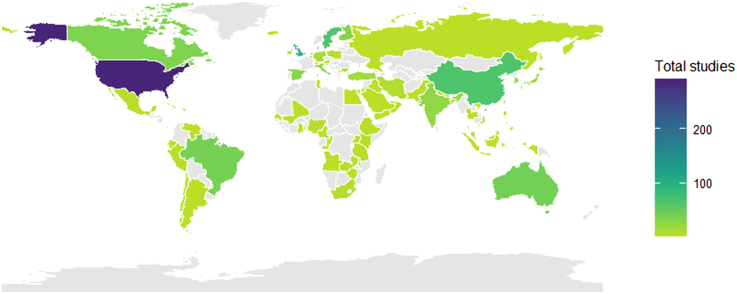
Total number of meta-analyses by country. Choropleth map showing the absolute number of meta-analyses by country. Darker shading indicates a higher number of included studies.

There were 1409 primary studies included in the meta-analyses. It was not possible to identify the country of the primary study in 281 cases. The remaining 1128 primary studies were conducted across 77 countries, including: 905 primary studies conducted in high income countries, 150 upper middle income, 60 lower middle income and 13 lower income (Ethiopia n = 3, Malawi n = 3, Mali n = 2, Togo n = 1, Uganda n = 3, Yemen n = 1). Individual countries contributing the greatest number of primary studies were: the United States of America (n = 290), the United Kingdom (n = 96), China (n = 65) and Sweden (n = 65) (Fig. 2).

Overall, 45 (34%) meta-analyses were classified as having convincing (7%) or highly suggestive (27%) evidence for the association of disability and the health condition (Table 1; Table 2; Supplementary Table S5). People with disabilities generally experience twice the frequency of health problems based on the outcomes where the research evidence is strongest. Across these outcomes, most pooled effect sizes ranged between a 1.5- to 3-fold higher odds or risk of adverse health conditions among people with disabilities compared with those without, with the largest effects observed for epilepsy, respiratory infections, and perinatal mortality (up to eightfold risk). The strength of evidence of an association with disability was highest for specific health outcome categories, infectious disease, perinatal and metabolicoutcomes. Strong and consistent evidence also emerged for psychosocial disabilities (such as schizophrenia, bipolar disorder, and depression), which were associated with increased risks of physical health conditions and mortality (Table 2).

**Table 2:** Effect sizes across included studies.

Intellectual and developmental disabilities were consistently associated with higher risks of epilepsy, respiratory infections, obesity, diabetes, and adverse perinatal outcomes, often supported by highly suggestive or convincing evidence. Psychosocial disabilities were associated to higher risks of metabolic disorders, pregnancy complications, and mortality, with moderate evidence of associations with cardiovascular disease. Intellectual and developmental disabilities were associated to higher risks of epilepsy, respiratory infections, obesity, diabetes, and adverse perinatal outcomes, often supported by highly suggestive or convincing evidence. Hearing and vision impairments were associated with greater risks of depression, cognitive decline or dementia, stroke, and mortality, typically based on suggestive to convincing findings.

Of the 132 meta-analyses, only 19 (14%) did not find a positive and statistically significant association between disability and the health conditions (95% confidence intervals excluding the null value). There is sparse and non-significant evidence for the association of disability with the health outcomes categories of neoplasms, diseases of the visual system and diseases of the digestive system. No studies were identifed on disability in relation to diseases of the blood, diseases of the immune system, diseases of the musculoskeletal system or conditions related to sexual health.

**Fig. 3:**
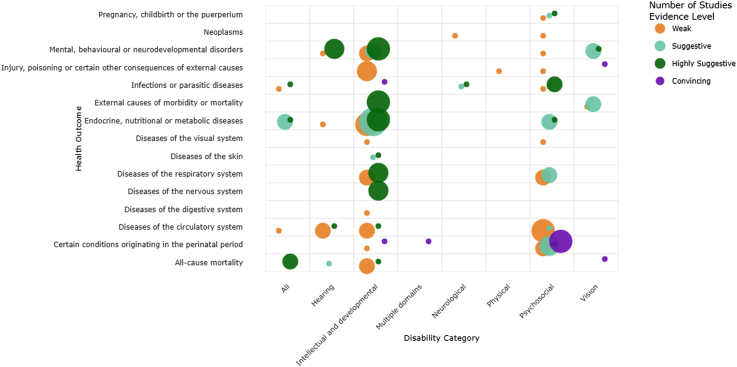
Map of meta-analyses of disability and ICD-11 codes. Bubble plot showing the number of meta-analyses (bubble size) across disability categories and ICD-11 health outcomes. Colour indicates the strength of evidence (weak, suggestive, highly suggestive, or convincing) based on the predefined criteria.

There is variability in the amount and strength of the evidence by ICD-11 health outcomes and disability categories (Fig. 3). For example, endocrine, nutritional or metabolic diseases and all-cause mortality are supported by multiple meta-analyses with strong evidence. Among disability categories, the greatest density and strongest evidence is found for intellectual and developmental disabilities followed by psychosocial disabilities. Other health outcomes such as neoplasms and diseases of the digestive system, and neurological and physical disabilities demonstrate notable gaps in the availability of meta-analyses.

Following the updated search, after deduplication and screening according to the original eligibility criteria, four additional studies met inclusion and are cited in the reference list.^82^, ^83^, ^84^, ^85^ The inclusion of these studies did not change the direction or strength of the main findings, and details of the studies are provided in Supplementary Table S6.

## Discussion

Our umbrella review highlights that people with disabilites experience vast health inequalities across the lifecourse, diseases, and disability types. Almost all meta-analyses (n = 113, 86%) found statistically significant and positive associations between disability and the health outcome, and this was deemed to be convincing or suggestive evidence for one third of analyses. Overall, positive associations were observed across disability types, ages, and health outcome areas. People with disabilities generally experience double the frequency of health conditions, based on the outcomes where the research evidence is strongest. The strongest associations were found with mortality (including all-cause and COVID-19), metabolic diseases (e.g., diabetes and obesity), perinatal outcomes (e.g., preterm birth, stillbirth) and mental ill health conditions (e.g., depression, anxiety, suicidal ideation). Of these, the large effect sizes observed for mental ill health outcomes likely reflect the bidirectional relationship between disability and psychological distress. People with disabilities may experience social exclusion, stigma, discrimination, pain, and functional limitations, which can exacerbate anxiety and depression. Limited opportunities for employment and social participation, environmental barriers, and reduced autonomy can further heighten these adverse mental ill health outcomes. In addition, the lack of disability-inclusive mental health services and inadequate integration of psychosocial support into disability care pathways contribute to this increased frequency.^86^^,^^87^

This service fragmentation is mirrored in the research evidence itself. There was great variability in the amount of data available by category–nearly half of the studies (45%) related to people with intellectual and developmental disabilities and there was limited data on multiple disabilities, which is surprising given how often impairments co-occur. Similarly, domains such as injury prevention, infectious diseases, and surgical outcomes are known areas of risk and disparity, yet were rarely studied in meta-analyses. There were no studies on disability in relation to diseases of the blood, diseases of the immune system, diseases of the musculoskeletal system or conditions related to sexual health. There were few meta-analyses that were exclusive to older adults, despite their high prevalence of disability. These results highlight the large gaps in our understanding of health for 16% of the worlds’ population, with 80% of the meta-analyses published only in the last five years.

Our findings are consistent with previous reports. A scoping review undertaken in 2014^88^ identified only 29 studies and reported that the most frequently examined diseases in relation to disability were diabetes and heart disease. Since then, there has been growing global recognition of the need to address health inequalities among people with disabilities, although evidence gaps persist. A more recent scoping review published in 2025 identified 51 studies and demonstrated that people with disabilities experience persistent and widespread health inequities across access to healthcare and resourcesand social determinants of health.^89^ Other reviews describe the barriers and health system failures that contribute to these health inequities.^3^^,^^90^ These findings align with the WHO Global report on health equity for persons with disabilities,^4^ which highlights that these inequities are avoidable and unjust, and calls for urgent, inclusive action across all health systems.

This umbrella review sheds light on the uneven landscape of evidence and calls for greater equity in research, both in terms of the types of disability included and the breadth of health outcomes considered, as well as the need for more studied to be undertaken in LMICs. Meta analyses should be undertaken to fill key evidence gaps—such as disability in relation to blood, immune, musculoskeletal, or sexual health conditions. New primary studies are potentially also needed to fill gaps in evidence, particularly for underrepresented health conditions (e.g. sexual health), and to expand beyond the current focus on intellectual and developmental disabilities. The routine inclusion of validated disability measures in national health surveys, electronic health records, and longitudinal studies is essential to ensure more health data on disability. Standardising disability definitions and improving cross-country comparability would enhance monitoring of disparities. Additionally, equity-focused indicators should be embedded in health system performance metrics to track progress and hold systems accountable for reducing gaps in care and outcomes.^3^ Studies should also consider differences within specific ICD-11 categories. For example, within neoplasms, some cancers are considered preventable (e.g., cervical, lung), whilst others are highly treatable if detected early (e.g., breast, colorectal).^91^ Analysing all cancers as a single category may obscure disparities in access to prevention, early detection, and treatment. Stratifying meta-analyses by cancer type, particularly by preventability and treatability, would provide clearer insight into where inequities lie. Our umbrella review also identified key ways in which the conduct and reporting of systematic reviews and meta-analyses need to be improved, to strengthen the evidence. Notably, the overall number of participants included in the meta-analyses was often not reported. In addition, the meta-analyses often showed a high heterogeneity (I^2^ > 50%). Finally, future studies should aim to understand the interventions that would improve these inequities in health outcomes, as well as the impact on individuals’ quality of life.

Our study has limitations that need to be considered when interpreting the results. First, these results only include meta-analyses, and so will overlook individual studies on a particular topic for which a meta-analysis is lacking (e.g., sexual and reproductive health outcomes), and systematic reviews would have been excluded if no meta-analysis was undertaken. Second, as some conditions lead to disability only when associated impairments interact with contextual barriers, our criteria may have excluded studies where this distinction was not clearly made. Additionally, this review only considers the literature on outcome measures of health, while self-rated health and health-related quality of life are also important to measure. It also cannot assess the amount of the health inequality that is avoidable, and thus the extent of health inequities, for instance through improved healthcare access. Nevertheless, this umbrella review identified 58 systematic reviews that assessed association of disability and health outcomes that contained mostly high-quality individual studies. Strengths of this approach include undertaking a gold standard approach to the umbrella review, capturing a broad range of conditions and health outcomes, and categorisation of the strength of the evidence.

Our findings have important implications for health policy and practice and highlight the need to explicitly address the systemic inequities experienced by people with disabilities. The evidence base needs to be strengthened on how to improve disability inclusion in the health system. A systems approach is needed, embedding disability inclusion across all elements of health system strengthening—governance, financing, workforce, service delivery, and information systems, and linking policy commitments with practice. This approach requires coordinated action at multiple levels. At the policy and governance level, key priorities include integrating disability-specific indicators into national health monitoring systems to track disparities and inform targeted action; mandating that all health policies and programmes explicitly consider and address the needs of people with disabilities to ensure disability-inclusive design and implementation; and allocating dedicated funding for disability-inclusive health services, such as accessible infrastructure and assistive technologies. At the service level, building a disability-inclusive health system also requires attention to practice-level changes, such as training healthcare workers on disability and providing reasonable adjustments in service delivery models to ensure physical, sensory, and cognitive accessibility, for example, through extended appointment times and easy read information formats. Equally important is involving people with disabilities in the design and evaluation of health services to ensure care is responsive to their needs.

People with disabilities are highly diverse, yet are unified in the experience of profound health inequities. Health systems must act to build equitable, disability-inclusive care that leaves no one behind. With less than a decade to meet various global health goals, evidence on the health disparities experienced by people with disabilities and interventions to close these gaps are urgently required. It is essential to understand and quantify these gaps, as it is impossible to act without evidence and illuminating these inequities.

## Contributors

TS, SR, JW and HK conceived the study and developed the research protocol. TS and SR developed the data collection instruments, and co-led data collection. TS, SR and MMC extracted data. TS led analysis and development of figures. TS wrote the first draft of the manuscript with input from SR, JW, MMC and HK. All authors approved of the final version for publication. TS, SR and MMC had full access to and verify all data in the study.

## Data sharing statement

No additional data are available.

## Editorial note

The *Lancet* Group takes a neutral position with respect to territorial claims in published maps and institutional affiliations.

## Declaration of interests

All authors declare no competing interests.
