# Supplementary material for: Health inequalities among people with disabilities: an umbrella review and evidence synthesis
Source: eClinicalMedicine. 2025 Dec 18;91:103675. doi: 10.1016/j.eclinm.2025.103675 (PMC12775867; doi:10.1016/j.eclinm.2025.103675)
Supplement: Supplemental File [file mmc1.pdf]

Contents

S1: Search strategies 2
S2: Classification of evidence in meta-analysis for effect sizes 6
S3: Excluded full texts and reasons for exclusion 7
S4: AMSTAR2 scoring of included texts 9
S5: Classification of effect sizes 11
S6: Studies published between February and October 2025 18

S1: Search strategies

EMBASE

Disability	1	exp Disabled Person/
	2	((people or person* or adult* or men or women or child* or youth or adolescent* or girl* or boy*) adj1 disab*).ti,ab.
	3	((people or person* or adult* or men or women or child* or youth or adolescent* or girl* or boy*) adj1 handicap*).ti,ab.
	4	((people or person* or adult* or men or women or child* or youth or adolescent* or girl* or boy*) adj1 impair*).ti,ab.
	5	(special adj1 need*).ti,ab.
	6	(physical* adj1 (disab* or handicap* or impair* or abnormal* or defect*)).ti,ab.
	7	((congenital or genetic) adj1 (disab* or handicap* or impair* or abnormal* or defect*)).ti,ab.
	8	Cerebral Palsy/
	9	cerebral palsy.ti,ab.
	10	Spinal Dysraphism/
	11	spinal dysraphism*.ti,ab.
	12	spina bifida.ti,ab.
	13	Muscular Disorders, Atrophic/
	14	(muscular adj1 (abnormal* or disorder* or condition* or disab* or impair* or dystroph*)).ti,ab.
	15	Osteogenesis Imperfecta/
	16	Osteogenesis Imperfecta.ti,ab.
	17	Musculoskeletal Abnormalities/
	18	(musculoskeletal adj1 (abnormal* or disorder* or condition* or disab* or impair*)).ti,ab.
	19	Amyotrophic lateral sclerosis.ti,ab.
	20	ALS.ti,ab.
	21	Motor Neuron Disease/
	22	motor neurone disease.ti,ab.
	23	MND.ti,ab.
	24	Dementia/
	25	dementia.ti,ab.
	26	amput*.ti,ab.
	27	Clubfoot/
	28	clubfoot.ti,ab.
	29	Ataxia/
	30	ataxia.ti,ab.
	31	Poliomyelitis/
	32	polio*.ti,ab.
	33	Paralysis/
	34	paraly*.ti,ab.
	35	Paraplegi*.ti,ab.
	36	quadriplegi*.ti,ab.
	37	Hemiplegi*.ti,ab.
	38	(spin* adj1 injur*).ti,ab.
	39	wheelchair user*.ti,ab.
	40	Albinism/
	41	albin*.ti,ab.

	42	Dwarfism/
	43	Achondroplasia.ti,ab.
	44	dwarf*.ti,ab.
	45	(restricted adj1 (growth or stature)).ti,ab.
	46	Microcephaly.ti,ab.
	47	hearing loss/
	48	hearing disorders/
	49	(hearing adj1 (loss or disab* or handicap* or impair* or abnormal* or defect*)).ti,ab.
	50	(hard adj2 hearing).ti,ab.
	51	deaf*.ti,ab.
	52	persons with hearing impairments/
	53	Vision Disorders/
	54	(vision adj1 (disab* or handicap* or impair* or abnormal* or defect*)).ti,ab.
	55	(visual* adj1 (disab* or handicap* or impair* or abnormal* or defect*)).ti,ab.
	56	(blind not single blind* not double blind* not triple blind* not blinding not blinded not blindly).ti,ab.
	57	partial* sight*.ti,ab.
	58	Intellectual Disability/
	59	(intellectual* adj1 (disab* or handicap* or impair* or abnormal* or defect*)).ti,ab.
	60	learning disabilities/
	61	(learning adj1 (disab* or handicap* or impair* or abnormal* or defect*)).ti,ab.
	62	Developmental Disabilities/
	63	(development* adj1 (disab* or handicap* or impair* or abnormal* or defect*)).ti,ab.
	64	child development disorders, pervasive/
	65	autis*.ti,ab.
	66	f?etal alcohol syndrome.ti,ab.
	67	fragile x syndrome.ti,ab.
	68	down* syndrome.ti,ab.
	69	asperger*.ti,ab.
	70	rett syndrome.ti,ab.
	71	heller* syndrome.ti,ab.
	72	pervasive development* disorder*.ti,ab.
	73	attention deficit hyperactivity disorder.ti,ab.
	74	ADHD.ti,ab.
	75	neurodivers*.ti,ab.
	76	Communication Disorders/
	77	(communicat* adj1 (disab* or handicap* or impair* or abnormal* or defect*)).ti,ab.
	78	(speech adj1 (disab* or handicap* or impair* or abnormal* or defect*)).ti,ab.
	79	(language adj1 (disab* or handicap* or impair* or abnormal* or defect*)).ti,ab.
	80	Dysarthria.ti,ab.
	**81**	**1 or 2 or 3 or 4 or 5 or 6 or 7 or 8 or 9 or 10 or 11 or 12 or 13 or 14 or 15 or 16 or 17 or 18 or 19 or 20 or 21 or 22 or 23 or 24 or 25 or 26 or 27 or 28 or 29 or 30 or 31 or 32 or 33 or 34 or 35 or 36 or 37 or 38 or 39 or 40 or 41 or 42 or 43 or 44 or 45 or 46 or 47 or 48 or 49 or 50 or 51 or 52 or 53 or 54 or 55 or 56 or 57 or 58 or 59 or 60 or 61 or 62 or 63 or 64 or 65 or 66 or 67 or 68 or 69 or 70 or 71 or 72 or 73 or 74 or 75 or 76 or 77 or 78 or 79 or 80 [DISABILITY TERMS]**
Health terms	82	(Chronic adj1 (disease* OR ill* OR condition*)). ti, ab.
	83	(Noncommunicable disease* OR NCD* OR non-communicable condition*).ti,ab.
	84	exp health/
	85	(health problem OR health condition).ti, ab.

	86	(Infect* disease* OR communicable disease* OR infection* or disease transmission).ti,ab.
	87	exp “bacterial infections and mycoses”/ or exp virus diseases/ or exp parasitic diseases/
	88	exp musculoskeletal disease/ or exp digestive system diseases/ or exp respiratory tract diseases/ or exp nervous system diseases/ or exp "hemic and lymphatic diseases"/ or exp "skin and connective tissue diseases"/ or exp "nutritional and metabolic diseases"/ or exp endocrine system diseases/ or exp immune system diseases/
	89	(Maternal health OR maternal mortalit* OR maternal morbidit* OR (pregnancy adj2 condition*)).ti,ab.
	90	(gestational diabetes or gestational hypertension or preeclampsia or pregnancy complication* or delivery complication or postpartum hemorrhage or postpartum infection or postpartum complication*).ti,ab.
	91	(Child health OR child morbidity OR child mortality OR p?ediatric health OR neonatal health).ti,ab.
	92	exp Mortality/
	93	(Death$ OR fatality OR fatalities).ti, ab
	94	exp cardiovascular disease/
	95	((hypertension OR stroke OR heart disease OR ("myocardial infarction*" OR "heart attack*" OR “acute coronary syndrome” OR "myocardial ischemia" OR "atherosclerosis" OR “coronary artery disease” OR “coronary vascular disease” OR “coronary heart disease” OR "cerebrovascular disease*" OR "cerebral artery disease*" OR "cerebral ischemia" OR "cerebrovascular accident*" OR "brain infarction*" OR "brain ischemia" OR "cerebrovascular attack*" OR "peripheral arterial disease" OR "peripheral artery disease" OR "peripheral vascular disease")).ti,ab.
	96	exp diabetes/
	97	("Type I Diabetes Mellitus" OR "Insulin Dependent Diabetes Mellitus"). ti, ab.
	98	exp cancer/
	99	(Neoplasm* OR Neoplasia* OR Tumor* OR Tumour* OR Cancer* OR Malignanc*).ti,ab.
	100	exp respiratory disease/
	101	(tuberculosis OR TB OR HIV OR AIDS OR malaria OR hepatitis OR COVID OR dengue OR zika OR ebola).ti, ab.
	102	HIV infections/ OR HIV seropositivity/
	103	(HIV OR Human Immunodeficiency Virus OR HTLVIII OR HTLV III OR HTLV3).ti,ab.
	104	exp suicide/
	105	exp malnutrition/
	106	(anthropometric failure or malnourish* or malnutrition or wast* or undernutrition or undernourished or marasm* or kwashiorkor or stunt* or underweight or severe acute malnutrition or SAM or body mass index or BMI or MUAC or mid-upper arm circumference or mid upper arm circumference).ti,ab.
	107	exp Protein-Energy Malnutrition/or Malnutrition/or child malnutrition/or infant malnutrition/
	108	exp Dementia/
	109	exp alzheimer's disease/
	110	(mild cognitive impairment).ti, ab.
	111	exp Hospital admission/
	112	(inpatient OR secondary care).ti,ab.
	113	exp Epilepsy/
	114	(epilep* OR seizure* OR convulsi*).ti,ab.
	115	exp health care quality/
	116	((access* or equal* or inequal* or barrier* or afford* or accept* or avail* or prevent* or treat* or diagn* or us*1 or usage or utili#ation or right* or disparit* or coverage or universal) adj3 (health or healthcare)).mp.
	117	health care delivery/ or exp health care access/ or exp universal health care/

	118	exp health care utilization/
	119	((clinical governance or evaluation* or qualit* or standard* or patient* need* or patient* satisfaction* or experience* or preference* or need* or satisfaction* or people-centredness or patient-centred or patient centered or attitude* or skill* or knowledge or responsiveness) adj2 (health or healthcare)).ti,ab.
	120	((plan* or insurance* or program* or benefit* or expenditure* or "out-of-pocket payment*" or "financial risk protection") adj3 (health or medical)).ti,ab.
	121	exp health insurance/
	122	program* acceptabilit*.ti,ab.
	123	exp program acceptability/
	**124**	**82 or 83 or 84 or 85 or 86 or 87 or 88 or 89 or 90 or 91 or 92 or 93 or 94 or 95 or 96 or 97 or 98 or 99 or 100 or 101 or 102 or 103 or 104 or 105 or 106 or 107 or 108 or 109 or 110 or 111 or 112 or 113 or 114 or 115 or 116 or 117 or 118 or 119 or 120 or 121 or 122 or 123 [ALL HEALTH TERMS]**
Study type	125	exp Meta-Analysis as Topic/
	126	(meta-analys* or meta analys*).ti,ab.
	127	**125 or 126 [STUDY TERMS]**
ALL TERMS	128	81 AND 124 AND 127

Medline and PsycINFO

MEDLINE term	PsycINFO equivalent term
exp Disabled Persons/	**exp Physical Disabilities/**
exp Hearing Loss/	**exp Hearing Impairments/**
exp Vision Disorders/	**exp Visual Impairments/**
exp Intellectual Disability/	**exp Intellectual Development Disorders/**
exp Developmental Disabilities/	exp Developmental Disabilities/
exp Communication Disorders/	exp Communication Disorders/
exp Health/	**exp Physical Health/**
exp Mortality/	**exp Death/**
exp Diabetes Mellitus/	**exp Diabetes/**
exp Neoplasms/	**exp Cancer/**
exp Health Services Accessibility/	**exp Health Care Services/**
exp Meta-Analysis as Topic/	**exp Meta Analysis/**

Cochrane Library and Health Evidence

1	Disability OR disabilities OR people with disabilities OD disabled person
2	Health
3	1 AND 2

S2: Classification of evidence in meta-analysis for effect sizes

Convincing evidence (class 1)
- ≥1000 cases, or ≥100,000 overall sample size
- $p \leq 10^{-6}$ for effect model
- Low to moderate between-studies heterogeneity ($I^2 \leq 50\%$)
- 95% CIs excluding the null value
- No evidence of publication bias

Highly suggestive evidence (class 2)
- ≥1000 cases, or ≥100,000 overall sample size
- $p \leq 10^{-6}$ for effect model
- Class 1 was not met

Suggestive evidence (class 3)
- ≥1000 cases, or ≥100,000 overall sample size
- $p \leq 10^{-3}$ for effect model
- Class 2 was not met

Weak evidence (class 4)
- $p \leq 0{\cdot}05$

Non-significant evidence
- $p > 0{\cdot}05$

S3: Excluded full texts and reasons for exclusion

Author (Year)	Title	Exclusion reason
Andiwijaya (2022)	Disability and Participation in Breast and Cervical Cancer Screening: A Systematic Review and Meta-Analysis.	No/wrong health outcome
Asiri (2024)	Oral health status of children with autism spectrum disorder in KSA: A systematic review and meta-analysis	No/wrong health outcome
Bakhla (2023)	Prevalence of depression in visually impaired children and adolescents: A systematic review and meta-analysis	Prevalence with no comparator
Bellato (2023)	Association between ADHD and vision problems. A systematic review and meta-analysis.	No/wrong health outcome
Bird (2020)	Dental caries experience, care index and restorative index in children with learning disabilities and children without learning disabilities: a systematic review and meta-analysis.	No/wrong health outcome
Chalk (2016)	Rates of Type 2 diabetes, cardiovascular disease and associated risk factors in people with intellectual disability populations: Systematic review and meta-analysis	Prevalence with no comparator
Crowder (2022)	Is the oral health of visually impaired children and adolescents different compared to their sighted peers?.	No/wrong health outcome
Deierlein (2021)	Pregnancy-related outcomes among women with physical disabilities: A systematic review	No/wrong health outcome
Deierlein (2024)	Mental health outcomes across the reproductive life course among women with disabilities: A systematic review.	No/wrong health outcome
Drumond (2022)	Dental Caries in Children with Attention Deficit/Hyperactivity Disorder: A Meta-Analysis.	No/wrong health outcome
Edwards (2022)	Prevalence of anxiety symptomatology and diagnosis in syndromic intellectual disability: A systematic review and meta-analysis.	Prevalence with no comparator
Foley (2014)	Death resulting from pneumonia is increased in dementia: A systematic review and meta-analysis	No/wrong health outcome
Garas (2020)	Long-term suicide risk of children and adolescents with attention deficit and hyperactivity disorder-A systematic review.	No/wrong health outcome
Glinianaia (2020)	Long-term survival of children born with congenital anomalies: A systematic review and meta-analysis of population-based studies.	No/wrong health outcome
Gu (2023)	The Association Between Congenital Heart Disease and Autism Spectrum Disorder: A Systematic Review and Meta-Analysis.	Prevalence with no comparator
Ho (2021)	Neonatal and infant mortality associated with spina bifida: A systematic review and meta-analysis.	Prevalence with no comparator
Hong (2019)	Visual impairment and mortality: Systematic review and meta-analysis with data from the EPIC-norfolk eye study	No/wrong health outcome
Hossain (2020)	Prevalence of comorbid psychiatric disorders among people with autism spectrum disorder: An umbrella review of systematic reviews and meta-analyses.	Prevalence with no comparator
Kakuszi (2023)	[Self-harming behavior and suicide attempts in autism spectrum disorder: A systematic overview].	Prevalence with no comparator
Kwon (2024)	Multipsychiatric Comorbidity in People With Epilepsy Compared With People Without Epilepsy: A Systematic Review and Meta-analysis.	No/wrong health outcome
Lam (2020)	Oral health status of children and adolescents with autism spectrum disorder: A systematic review of case-control studies and meta-analysis.	No/wrong health outcome
McCormick (2021)	Experiences of adults with intellectual disabilities accessing acute hospital services: A systematic review of the international evidence.	Measurement of healthcare access
McIntyre (2012)	Increased mortality among older adults after a traumatic brain injury: A meta-analysis	No/wrong health outcome
Mocanu (2019)	The impact of ADHD on outcomes following bariatric surgery: A systematic review and meta-analysis	No/wrong health outcome
Park (2023)	Prevalence and Mortality Risk of Neurological Disorders during the COVID-19 Pandemic: An Umbrella Review of the Current Evidence.	No/wrong health outcome

Pinquart (2020)	Health-Related Quality of Life of Young People With and Without Chronic Conditions.	No/wrong health outcome
Power (2024)	Cervical screening participation and access facilitators and barriers for people with intellectual disability: a systematic review and meta-analysis	No/wrong health outcome
Putri (2021)	Parkinson's disease may worsen outcomes from coronavirus disease 2019 (COVID-19) pneumonia in hospitalized patients: A systematic review, meta-analysis, and meta-regression.	No/wrong health outcome
Rahmati (2024)	Factors Affecting Global Adherence for the Uptake of Diabetic Retinopathy Screening: A Systematic Review and Meta-Analysis.	No/wrong health outcome
Ricciardi (2024)	Disability and Participation in Colorectal Cancer Screening: A Systematic Review and Meta-Analysis.	No/wrong health outcome
Rydzewska (2016)	Co-morbid health conditions in people with autism spectrum disorder: A systematic review of systematic reviews and meta-analyses	Prevalence with no comparator
Shang (2021)	The Association between Vision Impairment and Incidence of Dementia and Cognitive Impairment: A Systematic Review and Meta-analysis.	No/wrong health outcome
Silva (2020)	Caries experience in children and adolescents with Down Syndrome: A systematic review and meta-analysis.	Wrong health outcome
Suffel (2022)	Exploring the impact of mental health conditions on vaccine uptake in high-income countries	No/wrong health outcome
Tosetti (2023)	Do people with disabilities experience disparities in cancer care? A systematic review.	Measurement of healthcare access
Treskova-Schwarzbach (2021)	Pre-existing health conditions and severe COVID-19 outcomes: an umbrella review approach and meta-analysis of global evidence.	No/wrong health outcome
Trott (2021)	Hearing impairment and diverse health outcomes : An umbrella review of meta-analyses of observational studies.	No/wrong health outcome
Uliana (2024)	Autistic individuals have worse oral status than neurotypical controls: a systematic review and meta-analysis of observational studies.	Wrong health outcome
VanDerSchans (2016)	Association between atopic diseases and attention-deficit/hyperactivity disorder: Systematic review and meta-analyses	No/wrong health outcome
Wehrli (2023)	Quality of life of pediatric and adult individuals with osteogenesis imperfecta: a meta-analysis.	No/wrong health outcome
Wei (2017)	Hearing impairment, mild cognitive impairment, and dementia: A meta-analysis of cohort studies	No/wrong health outcome
Wei (2025)	Bidirectional association between allergic rhinitis and attention-deficit/hyperactivity disorder: A systematic review and meta-analysis.	No/wrong health outcome
Xu (2013)	Parkinson's disease and risk of mortality: Meta-analysis and systematic review	No/wrong health outcome
Zhang (2020)	Dental Caries Status in Autistic Children: A Meta-analysis.	No/wrong health outcome
Zheng (2017)	Hearing impairment and risk of Alzheimer's disease: a meta-analysis of prospective cohort studies.	No/wrong health outcome

S4: AMSTAR2 scoring of included texts

Questions: Q1: Did the research questions and inclusion criteria for the review include the components of PICO?; Q2: Did the report of the review contain an explicit statement that the review methods were established prior to the conduct of the review and did the report justify any significant deviations from the protocol?; Q3: Did the review authors explain their selection of the study designs for inclusion in the review?; Q4: Did the review authors use a comprehensive literature search strategy?; Q5: Did the review authors perform study selection in duplicate?; Q6: Did the review authors perform data extraction in duplicate?; Q7: Did the review authors provide a list of excluded studies and justify the exclusions?; Q8: Did the review authors describe the included studies in adequate detail?; Q9: Did the review authors use a satisfactory technique for assessing the risk of bias (RoB) in individual studies that were included in the review?; Q10: Did the review authors report on the sources of funding for the studies included in the review?; Q11: If meta-analysis was performed did the review authors use appropriate methods for statistical combination of results?; Q12: If meta-analysis was performed, did the review authors assess the potential impact of RoB in individual studies on the results of the meta-analysis or other evidence synthesis?; Q13: Did the review authors account for RoB in individual studies when interpreting/ discussing the results of the review?; Q14: Did the review authors provide a satisfactory explanation for, and discussion of, any heterogeneity observed in the results of the review?; Q15: If they performed quantitative synthesis did the review authors carry out an adequate investigation of publication bias (small study bias) and discuss its likely impact on the results of the review?; Q16: Did the review authors report any potential sources of conflict of interest, including any funding they received for conducting the review

**Author (Year)**	**Q1**	**Q2**	**Q3**	**Q4**	**Q5**	**Q6**	**Q7**	**Q8**	**Q9**	**Q10**	**Q11**	**Q12**	**Q13**	**Q14**	**Q15**	**Q16**
Adane (2021)[30]	Yes	Yes	Yes	Yes	Yes	Yes	Partially	Yes	Yes	No	Yes	Yes	Yes	Yes	No	Yes
Ai (2022)[29]	Yes	Yes	Yes	Yes	Yes	Partially	Partially	Yes	Yes	No	Yes	No	No	Yes	Yes	Yes
Alzahrani (2024)[30]	Yes	Yes	Yes	Yes	Yes	Partially	Partially	Yes	Yes	No	Yes	Yes	No	No	No	Yes
Arrondo (2022)[31]	Yes	Yes	Yes	Yes	Yes	Yes	Partially	Yes	Yes	No	Yes	Yes	Yes	No	No	Yes
Bensi (2020)[32]	Yes	Yes	Yes	Yes	Yes	Yes	Partially	Yes	Yes	No	Yes	Yes	Yes	Yes	Yes	Yes
Blanchard (2021)[33]	Yes	Yes	Yes	Yes	Yes	Partially	Partially	Yes	Yes	No	Yes	Yes	Yes	Yes	Yes	Yes
Brancati (2021)[34]	Yes	Yes	Yes	Yes	Yes	Partially	Partially	Yes	No	No	Yes	No	No	Yes	No	Yes
Cao (2023)[35]	Yes	Yes	Yes	Yes	Yes	Yes	Partially	Yes	Yes	No	Yes	No	No	Yes	Yes	Yes
Catala-Lopez (2015)[36]	Yes	Yes	Yes	Yes	Yes	Yes	Partially	Yes	Yes	No	Yes	No	Yes	Yes	Yes	Yes
Catala-Lopez (2022)[33]	Yes	Yes	Yes	Yes	Yes	Yes	Partially	Yes	Yes	No	Yes	Yes	Yes	Yes	Yes	Yes
Chan (2024)[34]	Yes	Yes	Yes	Yes	Yes	Yes	Partially	Yes	Yes	No	Yes	No	Yes	Yes	Yes	Yes
Cortese (2022)[35]	Yes	Yes	Yes	Yes	Yes	Yes	Partially	Yes	Yes	No	Yes	Yes	Yes	Yes	Yes	Yes
Dai (2024)[40]	Yes	Yes	Yes	Yes	Yes	Yes	Partially	Yes	Yes	No	Yes	No	Yes	Yes	Yes	Yes
de Beaudrap (2014)[41]	Yes	Yes	Yes	Yes	Yes	Partially	Partially	Yes	Yes	No	Yes	No	Yes	Yes	Yes	Yes
de Oliveira (2024)[42]	Yes	Yes	Yes	Yes	Yes	Yes	Partially	Yes	Yes	No	Yes	Yes	No	Yes	Yes	Yes
Dhanasekara (2023)[43]	Yes	Yes	Yes	Yes	Yes	Partially	Partially	Yes	Yes	No	Yes	No	No	Yes	No	Yes
Erlich (2021)[44]	Yes	Yes	Yes	Yes	Yes	Yes	Partially	Yes	Yes	No	Yes	Yes	Yes	Yes	Yes	Yes
Etchecopar-Erchart (2022)[45]	Yes	Yes	Yes	Yes	Yes	Yes	Partially	Yes	No	No	Yes	No	No	Yes	Yes	Yes
Garcia-Argibay (2023)[42]	Yes	Yes	Yes	Yes	Yes	Partially	Partially	Yes	Yes	No	Yes	No	Yes	Yes	Yes	Yes
Hartman (2023)[47]	Yes	Yes	Yes	Yes	Yes	Partially	Partially	Yes	No	No	Yes	No	No	Yes	Yes	Yes
Hume-Nixon (2018)[16]	Yes	Yes	Yes	Yes	Yes	No	Partially	Yes	Yes	No	Yes	No	No	Yes	No	Yes

Kahathuduwa (2022)[44]	Yes	Yes	Yes	Yes	Yes	No	Partially	Yes	Yes	No	Yes	Yes	Yes	Yes	Yes	Yes
Kang -ADHD (2024)[45]	Yes	Yes	Yes	Yes	Yes	Partially	Partially	Yes	No	No	Yes	No	No	Yes	Yes	Yes
Kang - BD (2024)[50]	Yes	Yes	Yes	Yes	Yes	Yes	Partially	Yes	Yes	No	Yes	No	Yes	Yes	Yes	Yes
Kim (2024)[51]	Yes	Yes	Yes	Yes	Yes	Yes	Partially	Yes	No	No	Yes	No	No	Yes	Yes	Yes
Kuper (2023)[52]	Yes	Yes	Yes	Yes	Yes	Partially	Partially	Yes	Yes	No	Yes	Yes	Yes	Yes	Yes	Yes
Kuper (2024)[53]	Yes	Yes	Yes	Yes	Yes	Partially	Partially	Yes	Yes	No	Yes	Yes	Yes	Yes	Yes	Yes
Kuzma (2021)[54]	Yes	Yes	Yes	Yes	Yes	Partially	Partially	Yes	Yes	No	Yes	Yes	Yes	Yes	No	Yes
Lam (2021)[51]	Yes	Yes	Yes	Yes	Yes	Yes	Partially	Yes	Yes	No	Yes	No	Yes	Yes	No	Yes
Lawrence (2020)[56]	Yes	Yes	Yes	Yes	No	Yes	Partially	Yes	Yes	No	Yes	Yes	No	Yes	Yes	Yes
Lee (2024)[57]	Yes	Yes	Yes	Yes	Yes	Yes	Partially	Yes	Yes	No	Yes	No	Yes	Yes	Yes	Yes
Liu (2021)[58]	Yes	Yes	Yes	Yes	Yes	Yes	Partially	Yes	Yes	No	Yes	Yes	No	Yes	Yes	Yes
Loughrey (2018)[17]	Yes	Yes	Yes	Yes	Yes	Yes	Partially	Yes	Yes	No	Yes	Yes	Yes	Yes	Yes	Yes
Mitra (2018)[59]	Yes	Yes	Yes	Yes	Yes	Yes	Partially	Yes	Yes	No	Yes	Yes	Yes	Yes	Yes	Yes
Nilchian (2023)[60]	Yes	Yes	Yes	Yes	Yes	Partially	Partially	Yes	Yes	No	Yes	No	Yes	Yes	Yes	Yes
Palbo (2023)[61]	Yes	Yes	Yes	Yes	Yes	Partially	Partially	Yes	Yes	No	Yes	Yes	Yes	Yes	Yes	Yes
Pan (2021)[62]	Yes	Yes	Yes	Yes	Yes	Partially	Partially	Yes	Yes	No	Yes	No	Yes	Yes	Yes	Yes
Pardamean (2022)[63]	Yes	Yes	Yes	Yes	Yes	Yes	Partially	Yes	Yes	No	Yes	No	Yes	Yes	Yes	Yes
Pi (2020)[64]	Yes	Yes	Yes	Yes	Yes	Yes	Partially	Yes	Yes	No	Yes	Yes	Yes	Yes	Yes	Yes
Salaeva (2020)[61]	Yes	Yes	Yes	Yes	Yes	Yes	Partially	Yes	Yes	No	Yes	No	Yes	Yes	No	Yes
Sammels (2021)[66]	Yes	Yes	Yes	Yes	Yes	Yes	Partially	Yes	No	No	Yes	No	No	Yes	No	yes
Santomauro (2024)[67]	Yes	Yes	Yes	Yes	Yes	Partially	Partially	Yes	No	No	Yes	No	No	No	Yes	Yes
Seens (2021)[64]	Yes	Yes	Yes	Yes	Yes	Partially	Yes	Yes	Yes	No	Yes	No	No	Yes	No	Yes
Shoham (2021)[70]	Yes	Yes	Yes	Yes	Partially	Partially	Partially	Yes	Yes	No	Yes	No	Yes	Yes	Yes	Yes
Silva -Freire (2022)[69]	Yes	Yes	Yes	Yes	Yes	Yes	Partially	Yes	Yes	No	Yes	Yes	Yes	Yes	No	Yes
Smythe (2024)[71]	Yes	Yes	Yes	Yes	Yes	Partially	Partially	Yes	Yes	No	Yes	Yes	Yes	Yes	Yes	Yes
Tan (2022)[72]	Yes	Yes	Yes	Yes	Yes	Yes	Partially	Yes	Yes	No	Yes	Yes	Yes	Yes	Yes	Yes
Tan (2024)[73]	Yes	Yes	Yes	Yes	Yes	Yes	Partially	Yes	Yes	No	Yes	No	Yes	No	Yes	Yes
Tarasoff (2020a)[74]	Yes	Yes	Yes	Yes	Yes	Yes	Partially	Yes	Yes	No	Yes	No	Yes	Yes	Yes	Yes
Tarasoff (2020b)[75]	Yes	Yes	Yes	Yes	Yes	Yes	Partially	Yes	Yes	No	Yes	No	Yes	Yes	Yes	Yes
Thomas (2022)[76]	No	Yes	Yes	Yes	Partially	Yes	Partially	No	Yes	No	Yes	No	No	No	No	No
Truesdale (2021)[77]	Yes	Yes	Yes	Yes	Yes	Partially	Partially	Yes	Yes	No	Yes	Yes	Yes	Yes	No	Yes
Trusinska (2024)[78]	Yes	Yes	Yes	Yes	Yes	Yes	Partially	Yes	Yes	No	Yes	Yes	Yes	Yes	No	Yes
Vai (2021)[79]	Yes	Yes	Yes	Yes	Yes	Yes	Partially	Yes	Yes	No	Yes	Yes	Yes	Yes	Yes	Yes
Vancampfort (2022)[80]	Yes	Yes	Yes	Yes	Partially	Partially	Partially	Yes	Yes	No	Yes	No	Yes	No	Yes	Yes
Zhang (2021)[81]	Yes	Yes	Yes	Yes	Yes	Yes	Partially	Yes	Yes	No	Yes	No	Yes	Yes	No	Partially
Zhang (2023)[82]	Yes	Yes	Yes	Yes	Yes	Partially	Partially	Yes	Yes	No	Yes	No	Yes	Yes	Yes	Yes
Zhu (2024)[83]	Yes	Yes	Yes	Yes	Yes	Yes	Partially	Yes	Yes	No	Yes	No	Yes	Yes	Yes	Yes

S5: Classification of effect sizes

First author (year)	Number of people with disabilities	Total sample	Effect size (95%CI)	p-value	p-value classification	I ²	Consideration of bias	Classification of evidence	Reason for classification
**Certain infectious or parasitic diseases**									
De Beaudrap (2014)	5,465	Not reported	RR: 1.31 (1.02 - 1.69)	0.036053698	≤0.05	84%	Yes	Weak	p value
Kang (2024)	Not reported	over 60 million	OR: 1.69 (1.21 - 2.37)	0.002216086	≤0.05	83%	Yes	Weak	p value
Kuper (2023)	4,981,302	Not reported	OR: 2.70 (2.40 - 3.20)	2.13×10	$\leq 1\times10^{-6}$	97%	Yes	Highly suggestive	I2
Liu (2021)	3,211,426	34,168,377	OR: 2.08 (1.61 - 2.69)	-3.00E+01	$\leq 1\times10^{-6}$	93%	Yes	Highly suggestive	I2
Pardamean (2022)	2,773	195,932	RR:2.22 (1.54 - 3.20)	1.92E-05	$\leq 1\times10^{-3}$	82%	Yes	Suggestive	p value
Thomas (2022)	Not reported	8,021,164	OR: 2.18 (1.63 - 2.90)	1.14E-07	$\leq 1\times10^{-6}$	Not reported	Yes	Highly suggestive	I2
Trusinska (2024)	Not reported	173,990	OR: 5.43 (3.02 - 9.76)	1.57E-08	$\leq 1\times10^{-6}$	14%	Yes	Convincing evidence	_
Trusinska (2024)	Not reported	173,990	OR: 6.14 (2.39 - 15.77)	1.63E-04	$\leq 1\times10^{-3}$	82%	Yes	Suggestive	p value
Vai (2021)	43,938	1,469,731	OR: 2.00 (1.58 - 2.54)	1.04E-08	$\leq 1\times10^{-6}$	93%	Yes	Highly suggestive	I2
**Neoplasms**									
Catala-Lopez (2015)	577,013	Not reported	OR: 0.92 (0.87 - 0.98)	0.006046142	≤0.05	95%	Yes	Weak	p value
Kang (2024)	Not reported	over 60 million	OR: 1.24 (0.98 - 1.59)	0.081434947	>0.05	92%	Yes	Non-significant	95%CI
Lee (2024)	164,242	Not reported	OR: 1.31 (1.04 - 1.65)	0.021830263	≤0.05	89%	Yes	Weak	p value
**Endocrine, nutritional or metabolic diseases**									
Ai (2022)	388,460	13,573,972	OR: 1.37 (1.17 - 1.61)	1.11E-04	$\leq 1\times10^{-3}$	64%	Yes	Suggestive	p value
Ai (2022)	388,460	13,573,972	OR: 2.05 (1.37 - 3.07)	4.88E-04	$\leq 1\times10^{-3}$	92%	Yes	Suggestive	p value
Arrondo (2022)	74,476	1,390,311	OR: 1.32 (1.18 - 1.47)	0.000000419	$\leq 1\times10^{-6}$	84%	Yes	Highly suggestive	I2
Arrondo (2022)	32,525	9,741,635	OR: 1.90 (1.51 - 2.39)	4.25E-08	$\leq 1\times10^{-6}$	94%	Yes	Highly suggestive	I2
Cortese (2022)	237,529	3,427,773	OR 1.53 (1.1 - 2.1)	0.009936775	≤0.05	95%	Yes	Weak	p value
Dhanasekara (2023)	276,173	8,009,479	RR: 1.64 (1.06 - 2.54)	0.026486705	≤0.05	86%	No	Weak	p value
Dhanasekara (2023)	276,173	8,009,479	RR: 1.57 (1.23 - 2.01)	3.18E-04	$\leq 1\times10^{-3}$	98%	No	Suggestive	p value

Dhanasekara (2023)	276,173	8,009,479	RR: 1.40 (1.02-1.93)	0.038618294	≤0.05	98%	No	Weak	p value
Etchecopar-Etchart (2022)	43,611	40,991,883	OR: 2.35 (1.57 - 3.52)	3.35E-05	$\leq 1\times10^{-3}$	80%	Yes	Suggestive	p value
Garcia-Argibay (2023)	103,022	5,738,287	OR: 2.29 (1.48 - 3.55)	2.05E-04	$\leq 1\times10^{-3}$	78%	Yes	Suggestive	p value
Hume-Nixon (2018)	1,312	2,506	OR: 2.97 (2.33 - 3.79)	2.11×10^{-18}	$\leq 1\times10^{-6}$	26%	No	Highly suggestive	Evidence of publication bias
Hume-Nixon (2018)	564	1,543	OR: 1.82 (1.40 - 2.36)	6.95E-06	$\leq 1\times10^{-3}$	60%	No	Suggestive	number of cases
Hume-Nixon (2018)	564	1,543	OR: 1.90 (1.32 - 2.75)	6.08E-04	$\leq 1\times10^{-3}$	68%	No	Suggestive	number of cases
Kahathuduwa (2022)	1,520	4,114	RR: 1.3 (0.78 - 2.1)	0.29907494	>0.05	63%	Yes	Non-significant	Confidence interval
Kang (2024)	Not reported	over 234 million	OR: 1.37 (0.91 - 2.07)	0.133221977	>0.05	>50%	No	Non-significant	Confidence interval
Kang (2024)	Not reported	over 234 million	OR: 2.29 (1.49 - 5.35)	0.011062221	≤0.05	>50%	No	Weak	p value
Kang (2024)	Not reported	over 234 million	OR: 1.55 (1.28 - 1.87)	5.85E-06	$\leq 1\times10^{-3}$	>50%	No	Suggestive	p value
Kang (2024)	Not reported	over 60 million	OR: 1.64 (1.30 - 1.99)	5.25E-06	$\leq 1\times10^{-3}$	96%	Yes	Suggestive	p value
Kang (2024)	Not reported	over 60 million	OR: 1.98 (1.55 - 2.52)	3.60E-08	$\leq 1\times10^{-6}$	77%	Yes	Highly suggestive	I2
Lam (2021)	3,034	11,890	OR: 1.88 (1.01 - 3.50)	0.046472048	≤0.05	79%	Yes	Weak	p value
Sammels (2022)	59,682	393,957	RR: 1.70 (1.44 - 1.99)	1.28E-10	$\leq 1\times10^{-6}$	90%	No	Highly suggestive	I2
Tarasoff (2020b)	Not reported	4,863,957	OR: 2.85 (0.79 - 10.31)	0.110007108	>0.05	94%	Yes	Non-significant	CI
Tarasoff (2020b)	Not reported	5,767,059	OR: 1.10 (0.76 - 1.58)	0.609707296	>0.05	86%	Yes	Non-significant	CI
Vancampfort (2022)	55,548	4,233,098	OR: 1.87 (1.40 - 2.48)	1.78E-05	$\leq 1\times10^{-3}$	93%	Yes	Suggestive	p value
Zhu (2024)	14981	143897	OR: 1.56 (1.32 - 1.85)	2.42E-07	$\leq 1\times10^{-6}$	88%	Yes	Highly suggestive	I2
**Mental, behavioural or neurodevelopmental disorders**									
Brancati (2021)	544	1,124	RR: 8.97 (4.26 - 18.87)	7.55E-09	$\leq 1\times10^{-6}$	54%	No	Weak	Number of participants

Cao (2023)	>7,000	76,373	OR: 1.61 (1.42 - 1.80)	3.55E-15	$\leq 1\times10^{-6}$	55%	No	Highly suggestive	I2
Hartman (2023)	550,748	15,097,562	OR: 4.95 (3.29 - 7.46)	1.89E-14	$\leq 1\times10^{-6}$	Not reported	No	Highly suggestive	I2
Hartman (2023)	550,748	15,097,562	OR: 4.51 (2.44 - 8.34)	1.55E-06	$\leq 1\times10^{-3}$	Not reported	No	Suggestive	p value
Hartman (2023)	550,748	15,097,562	OR: 8.7 (5.47 - 13.89)	1.3×10^{-19}	$\leq 1\times10^{-6}$	Not reported	No	Highly suggestive	I2
Hartman (2023)	550,748	15,097,562	OR: 4.6 (2.72 - 7.80)	1.36E-08	$\leq 1\times10^{-6}$	Not reported	No	Highly suggestive	I2
Kang (2024)	Not reported	over 234 million	OR: 5.88 (3.71 - 9.34)	5.42E-14	$\leq 1\times10^{-6}$	>50%	No	Highly suggestive	I2
Kang (2024)	Not reported	over 234 million	OR: 2.96 (1.74 - 5.04)	6.34E-05	$\leq 1\times10^{-3}$	>50%	No	Suggestive	p value
Kang (2024)	Not reported	over 234 million	OR: 3.00 (1.53 - 5.92)	0.001458829	≤ 0.05	>50%	No	Weak	p value
Kang (2024)	Not reported	over 234 million	OR: 1.83 (0.59 - 5.74)	0.297772677	>0.05	>50%	No	Non-significant	CI
Kuzma (2021)	3,415	37,705	RR: 1.38 (1.19 - 1.59)	1.32E-05	$\leq 1\times10^{-3}$	29%	Yes	Suggestive	p value
Lawrence (2020)	Not reported	147,148	OR: 1.47 (1.31 - 1.65)	5.96E-11	$\leq 1\times10^{-6}$	83%	Yes	Highly suggestive	I2
Lee (2024)	Not reported	66 million	OR: 2.29 (1.19 - 4.39)	0.012843934	≤ 0.05	99%	Yes	Weak	p value
Loughrey (2018)	1,395	6,825	OR 1.2 (1.01 - 1.4)	0.028612221	≤ 0.05	0%	No	Weak	p value
Shoham (2021)	≥ 1,614	≥ 26,600	OR: 1.76 (1.34 - 2.31)	4.72E-05	$\leq 1\times10^{-3}$	79%	Yes	Suggestive	p value
Zhang (2023)	47,853	Not reported	OR: 1.83 (1.42 - 2.24)	2.03E-07	$\leq 1\times10^{-6}$	86%	Yes	Highly suggestive	I2
Zhang (2023)	651,447	Not reported	OR: 1.43 (1.32 - 1.55)	3.3 × 10E-18	$\leq 1\times10^{-6}$	69%	Yes	Highly suggestive	I2
**Diseases of the nervous system**									
Pan (2021)	65,860	4,039,930	OR: 8.98 (5.08 - 15.40)	8.66E-15	$\leq 1\times10^{-6}$	96%	Yes	Highly suggestive	I2
Pan (2021)	65,860	4,039,930	OR: 12.42 (5.32 - 29.01)	5.81E-09	$\leq 1\times10^{-6}$	96%	Yes	Highly suggestive	I2
Pan (2021)	65,860	4,039,930	OR: 8.46 (6.32 - 11.32)	7.8 × 10E-47	$\leq 1\times10^{-6}$	98%	Yes	Highly suggestive	I2
**Diseases of the visual system**									

Kang (2024)	Not reported	over 234 million	OR: 1.93 (1.25 - 2.98)	0.003009895	≤0.05	>50%	No	Weak	p value
Shoham (2021)	≥ 7,955	≥ 2,488,400	OR: 1.85 (1.17 - 2.92)	0.008371396	≤0.05	89%	Yes		p value
**Diseases of the circulatory system**									
Dhanasekara (2023)	276,173	8,009,479	RR: 1.69 (1.20 - 2.40)	0.003002551	≤0.05	99%	No	Weak	p value
Dhanasekara (2023)	276,173	8,009,479	RR: 1.46 (1.42 - 1.50)	3.8×10^{-160}	$\leq 1\times10^{-6}$	0%	No	Highly suggestive	Publication bias
Dhanasekara (2023)	276,173	8,009,479	RR: 1.22 (0.98 - 1.52)	0.075743628	>0.05	99%	No	Non-significant	CI
Dhanasekara (2023)	276,173	8,009,479	RR: 1.19 (0.63 - 2.24)	0.590889842	>0.05	99%	No	Non-significant	CI
Etchecopar-Etchart (2022)	43,611	40991883	OR: 1.55 (1.02 - 2.36)	0.040566477	≤0.05	86%	Yes	Weak	p value
Kang (2024)	Not reported	over 234 million	OR: 0.91 (0.72 - 1.15)	0.429825998	>0.05	>50%	No	Non-significant	CI
Kang (2024)	Not reported	over 234 million	OR: 1.00 (0.70 - 1.43)	0.89	>0.05	>50%	No	Non-significant	CI
Kang (2024)	Not reported	over 60 million	OR: 1.28 (1.02 - 1.60)	0.031600649	≤0.05	32%	Yes	Weak	p value
Kang (2024)	Not reported	over 60 million	OR: 1.09 (0.89 - 1.34)	0.409069851	>0.05	69%	Yes	Non-significant	CI
Kang (2024)	Not reported	over 60 million	OR: 2.12 (0.74 - 6.02)	0.159972103	>0.05	82%	Yes	Non-significant	CI
Kang (2024)	Not reported	over 60 million	OR: 1.54 (0.83 - 2.87)	0.172486194	>0.05	81%	Yes	Non-significant	CI
Lee (2024)	Not reported	66 million	OR: 1.53 (1.12 - 2.11)	0.008487703	≤0.05	85%	Yes	Weak	p value
Lee (2024)	Not reported	66 million	OR: 1.71 (1.30 - 2.25)	1.26E-04	$\leq 1\times10^{-3}$	98%	Yes	Suggestive	p value
Lee (2024)	Not reported	66 million	OR: 1.81 (1.21 - 2.69)	0.003600721	≤0.05	71%	Yes	Weak	p value
Tan (2024)	Not reported	1,621,165	OR: 1.26 (1.16 - 1.37)	5.19E-08	$\leq 1\times10^{-6}$	78%	Yes	Highly suggestive	I2
Tan (2024)	Not reported	940,771	OR: 1.36 (1.13 - 1.64)	0.001212435	≤0.05	96%	Yes	Weak	p value
Tan (2024)	Not reported	4,680,349	OR: 1.38 (1.07 - 1.77)	0.012126976	≤0.05	99%	Yes	Weak	p value
Tarasoff (2020b)	Not reported	5,660,846	OR: 1.45 (1.16 - 1.82)	0.001222067	≤0.05	85%	Yes	Weak	p value
Tarasoff (2020b)	Not reported	4,864,028	OR: 2.84 (0.82 - 9.83)	0.099502609	>0.05	94%	Yes	Non-significant	CI

Tarasoff (2020b)	Not reported	6,021,857	OR: 1.77 (1.21 - 2.60)	0.003431672	≤0.05	87%	Yes	Weak	p value
**Diseases of the respiratory system**									
Arrondo (2022)	1,707,805	9741635	OR: 1.55 (1.17 - 2.06)	0.00253	≤0.05	100%	Yes	Weak	p value
Arrondo (2022)	1,707,805	9741635	OR: 1.36 (1.13 - 1.65)	0.00182	≤0.05	88%	Yes	Weak	p value
Dai (2023)	Not reported	729,375	OR: 1.46 (1.41 - 1.51)	1.6×10^{-98}	$\leq 1\times10^{-6}$	59%	Yes	Highly suggestive	I2
Kang (2024)	Not reported	over 60 million	OR: 1.86 (1.42 - 2.42)	5.04E-06	$\leq 1\times10^{-3}$	92%	Yes	Suggestive	p value
Lee (2024)	Not reported	66 million	OR: 1.71 (1.05 - 2.78)	0.030779976	≤0.05	Asthma: 85%	Yes	Weak	p value
Lee (2024)	Not reported	66 million	OR: 1.73 (1.25 - 2.37)	7.84E-04	$\leq 1\times10^{-3}$	98%	Yes	Suggestive	p value
Lee (2024)	Not reported	66 million	OR: 2.63 (1.11 - 6.23)	0.027993704	≤0.05	99%	Yes	Weak	P value
Mitra (2018)	Not reported	1,748,209	RR: 6.06 (4.93 - 7.45)	1.5×10^{-65}	$\leq 1\times10^{-6}$	65%	Yes	Highly suggestive	I2
Truesdale (2021)	Not reported	104,110	SMR: 10.86 (5.32 - 22.18)	5.81E-11	$\leq 1\times10^{-6}$	99%	Yes	Highly suggestive	I2
**Diseases of the digestive system**									
de Oliveira (2024)	435	903	OR: 1.1 (0.9 – 1.4)	0.3348	>0.05	58%	No	Non-significant	CI
Nilchian (2023)	1200	2435	OR: 0.91 (0.79 – 1.05)	0.18	>0.05	95%	Yes	Non-significant	CI
Pi (2020)	391	872	OR: 1.30 (1.02 – 1.66)	0.034	≤0.05	88%	Yes	Weak	p value
Silva-Freire (2022)	330	569	OR: 2.04 (0.89 - 4.68)	0.092233502	>0.05	83%	Yes	Non-significant	CI
**Diseases of the skin**									
Arrondo (2022)	1,707,805	9741635	OR: 1.41 (1.31 - 1.51)	2.6×10^{-21}	$\leq 1\times10^{-6}$	66%	Yes	Highly suggestive	I2
Arrondo (2022)	1,707,805	9741635	OR: 1.85 (1.35 - 2.55)	1.50E-04	$\leq 1\times10^{-3}$	92%	Yes	Suggestive	p value
**Pregnancy, childbirth or the puerperium**									
Etchecopar-Etchart (2022)	43,611	40991883	OR: 1.85 (1.52 - 2.25)	7.83E-10	$\leq 1\times10^{-6}$	57%	Yes	Highly suggestive	I2
Etchecopar-Etchart (2022)	43,611	40991883	OR: 2.28 (1.58 - 3.29)	1.06E-05	$\leq 1\times10^{-3}$	55%	Yes	Suggestive	p value

Etchecopar-Etchart (2022)	43,611	40991883	OR: 1.14 (1.04 - 1.24)	0.003498998	≤ 0.05	0%	Yes	Weak	p value
**Certain conditions originating in the perinatal period**									
Adane (2021)	Not reported	36,038,106	OR: 1.47 (1.28 - 1.68)	2.80E-08	$\leq 1\times10^{-6}$	79%	Yes	Highly suggestive	I2
Adane (2021)	Not reported	33,557,786	OR: 1.51 (1.28 - 1.79)	1.46E-06	$\leq 1\times10^{-3}$	73%	Yes	Suggestive	p value
Chan (2024)	Not reported	37,214,330	OR: 1.36 (1.14 - 1.63)	7.49E-04	$\leq 1\times10^{-3}$	51%	Yes	Suggestive	p value
Chan (2024)	Not reported	37,214,330	OR: 1.12 (1.04 - 1.21)	0.003343842	≤ 0.05	0%	Yes	Weak	p value
Chan (2024)	Not reported	37,214,330	OR: 1.62 (1.30 - 2.02)	1.78E-05	$\leq 1\times10^{-3}$	37%	Yes	Suggestive	p value
Chan (2024)	Not reported	37,214,330	OR: 1.41 (1.30 - 1.53)	2.22E-16	$\leq 1\times10^{-6}$	49%	Yes	Convincing evidence	_
Etchecopar-Etchart (2022)	43,611	40991883	OR: 2.06 (1.83–2.31)	3.96×10^{-35}	$\leq 1\times10^{-6}$	0%	Yes	Convincing evidence	_
Etchecopar-Etchart (2022)	43,611	40991883	OR: 1.41 (1.03–1.94)	3.48×10^{-2}	≤ 0.05	0%	Yes	Weak	p value
Etchecopar-Etchart (2022)	43,611	40991883	OR: 2.87 (2.11–3.89)	1.08×10^{-11}	$\leq 1\times10^{-6}$	0%	Yes	Convincing evidence	_
Etchecopar-Etchart (2022)	43,611	40991883	OR: 2.33 (1.81–3.01)	9.52×10^{-11}	$\leq 1\times10^{-6}$	15%	Yes	Convincing evidence	_
Salaeva (2020)	Not reported	19,238	OR: 2.03 (1.31 - 3.13)	0.001440117	≤ 0.05	0%	Yes	Weak	p value
Tarasoff (2020a)	Not reported	8,783,563	OR: 1.76 (1.59 - 1.96)	7.45×10^{-25}	$\leq 1\times10^{-6}$	27%	Yes	Convincing evidence	_
Tarasoff (2020a)	Not reported	11,191,691	OR: 1.37 (1.27 - 1.48)	6.66E-16	$\leq 1\times10^{-6}$	0%	Yes	Convincing evidence	_
**Symptoms, signs or clinical findings, not elsewhere classified**									
Kang (2024)	Not reported	over 60 million	OR: 0.76 (0.50 - 1.03)	0.136610035	>0.05	99%	Yes	Non-significant	CI
**Injury, poisoning or certain other consequences of external causes**									
Alzahrani (2024)	767	2,654	OR: 1.98 (1.51 - 2.59)	6.95E-07	$\leq 1\times10^{-6}$	73%	Yes	Weak	Participant number
Bensi (2020)	Not reported		OR: 2.29 (1.12 - 4.69)	0.023335515	≤ 0.05	77%	Yes	Weak	p value
Kang (2024)	Not reported	over 234 million	OR: 4.83 (0.52 - 9.14)	0.031276941	≤ 0.05	>50%	No	Weak	p value
Lee (2024)	Not reported	66 million	OR: 1.63 (1.10 - 2.40)	0.01409302	≤ 0.05	29%	Yes	Weak	p value

Seens (2021)	53,849	1,085,808	RR: 2.55 (not reported)	NA	>0.05	Not reported	Yes	Non-significant	CI
Silva-Freire (2022)	Not reported	1,204	OR: 3.86 (2.63 - 5.68)	7.22×10^{-12}	$\leq 1\times 10^{-6}$	0%	Yes	Convincing evidence	_
Zhang (2021)	103,833	500,115	OR: 1.17 (1.01 - 1.35)	0.033914205	≤0.05	98%	Yes	Weak	p value
**External causes of morbidity or mortality**									
Blanchard (2021)	Not reported	Not reported	OR: 3.26 (2.74 - 3.89)	2.91×10^{-39}	$\leq 1\times 10^{-6}$	93%	Yes	Highly suggestive	I2
Blanchard (2021)	Not reported	Not reported	OR: 3.18 (2.45-4.12)	2.03×10^{-18}	$\leq 1\times 10^{-6}$	85%	Yes	Highly suggestive	I2
Kang (2024)	Not reported	over 234 million	OR: 2.38 (1.79 - 3.15)	1.81E-09	$\leq 1\times 10^{-6}$	>50%	No	Highly suggestive	I2
Kim (2024)	Not reported	5,692,769	OR: 2.49 (1.71 - 3.63)	2.03E-06	$\leq 1\times 10^{-3}$	93%	Yes	Suggestive	p value
Kim (2024)	Not reported	5,692,769	OR: 1.89 (1.32 - 2.71)	5.22E-04	$\leq 1\times 10^{-3}$	74%	Yes	Suggestive	p value
Palbo (2024)	Not reported	292,121	OR: 2.62 (1.29 - 5.31)	0.007622591	≤0.05	Not reported	Yes	Weak	p value
Santomauro (2024)	Not reported	10.4 million	RR: 2.86 (2.06 - 4.03)	8.34E-10	$\leq 1\times 10^{-6}$	Not reported	No	Highly suggestive	I2
**All-cause mortality**									
Catala-Lopez (2022)	Not reported	642,260	RR: 2.37 (1.97 - 2.85)	4.72×10^{-20}	$\leq 1\times 10^{-6}$	89%	Yes	Highly suggestive	I2
Catala-Lopez (2022)	Not reported	642,260	RR: 2.13 (1.13 - 4.02)	0.019515503	≤0.05	98%	Yes	Weak	p value
Ehrlich (2021)	Not reported	446,088	HR: 1.29 (1.20 - 1.39)	1.11E-11	$\leq 1\times 10^{-6}$	31%	Yes	Convincing evidence	_
Kang (2024)	Not reported	over 234 million	OR: 2.13 (1.16 - 3.89)	0.014303	≤0.05	>50%	No	Weak	p value
Kuper (2024)	Not reported	Not reported	HR: 2.24 (1.84 - 2.72)	6.66E-16	$\leq 1\times 10^{-6}$	100%	Yes	Highly suggestive	I2
Smythe (2024)	270,571	Not reported	RR: 2.02 (1.77 - 2.30)	2.5×10^{-26}	$\leq 1\times 10^{-6}$	98%	Yes	Highly suggestive	I2
Tan (2022)	Not reported	1,213,756	HR: 1.13 (1.07 - 1.19)	6.57E-06	$\leq 1\times 10^{-3}$	77%	Yes	Suggestive	p value

S6: Studies published between February and October 2025

Primary author	Disability Category	Health Outcome(s)	Effect size (95%C)
Lindstedt[86]	Intellectual disability	Suicide mortality & self-harm	RR: 0.54 (0.33–0.89)<br>RR: 3.16 (2.30–4.35)
Lo[87]	Intellectual disability	Maternal & perinatal outcomes	RR gestational diabetes: 1.27 (1.10-1.46)<br>RR pre-eclampsia/eclampsia: 1.27 (1.10 – 1.46)<br>RR placenta abruption: 1.34 (1.11 – 1.62)
Vargas-Fernández[85]	Intellectual disability	Hypertension prevalence & treatment coverage	Prevalence ratio: 0.71 (0.47–1.05)<br>Prevalence ratio: 0.61 (0.47–0.81)
Zhang[88]	Hearing impairment	Maternal & neonatal outcomes	Gestational diabetes RR = 1.32 (95% CI 1.16–1.50)<br>Preeclampsia/eclampsia RR = 1.27 (95% CI 1.10–1.46)<br>Placental abruption RR = 1.34 (95% CI 1.11–1.62)
